# The directional migration and differentiation of mesenchymal stem cells toward vascular endothelial cells stimulated by biphasic calcium phosphate ceramic

**DOI:** 10.1093/rb/rbx028

**Published:** 2017-10-31

**Authors:** Ying Chen, Jing Wang, Xiangdong Zhu, Xuening Chen, Xiao Yang, Kai Zhang, Yujiang Fan, Xingdong Zhang

**Affiliations:** National Engineering Research Center for Biomaterials, Sichuan University, Chengdu 610064, China

**Keywords:** BCP ceramic, vascularization, BMSCs, VECs, migration, differentiation

## Abstract

Osteoinductivity of porous calcium phosphate (CaP) ceramics has been widely investigated and confirmed, and it might be attributed to the rapid formation of the vascular networks after *in vivo* implantation of the ceramics. In this study, to explore the vascularization mechanism within the CaP ceramics, the migration and differentiation of bone marrow-derived mesenchymal stem cells (BMSCs) under the stimulation of porous biphasic calcium phosphate (BCP) ceramic with excellent osteoinductivity were systematically investigated. The results indicated that the directional migration of BMSCs toward BCP ceramic occurred when evaluated by using a transwell model, and the BMSCs migration was enhanced by the seeded macrophages on the ceramic in advance. Besides, by directly culturing BMSCs on BCP ceramic discs under both *in vitro* and *in vivo* physiological environment, it was found that the differentiation of BMSCs toward vascular endothelial cells (VECs) happened under the stimulation of BCP ceramic, as was confirmed by the up-regulated gene expressions and protein secretions of VECs-related characteristic factors, including kinase insert domain receptor, von willebrand factor, vascular cell adhesion molecule-1 and cadherin 5 in the BMSCs. This study offered a possibility for explaining the origin of VECs during the rapid vascularization process after *in vivo* implantation of porous CaP ceramics and could give some useful guidance to reveal the vascularization mechanism of the ceramics.

## Introduction

Every year, millions of bone grafting procedures are performed worldwide, needing a large number of bone substitutes [1]. Nowadays, autografts and allografts are still the most common solutions for bone defects over the critical size in clinical application [2–4]. However, they have some disadvantages such as the finite resource, the additional invasive surgery, the risk of transmission of infectious diseases, and the immunological rejection by the host [3]. In such cases, artificially synthesized biomaterials for the repair of bone defects were proposed [4, 5]. Among the current synthetic bone-grafting substitutes, calcium phosphate (CaP) ceramics are promising candidates for clinical use. This is not only due to their excellent biocompatibility and osteoconductivity [6–8], but also their widely confirmed osteoinductivity [9–20]. However, the osteoinduction mechanism by CaP ceramics is so far unclear. It is well known that vascularization plays a key role in the healing process of bone fractures, and bone regeneration after a biomaterial implantation is highly dependent on the formation of abundant vascular networks within the implant [21–24]. Thus, the excellent osteoinductivity of CaP ceramics might be closely related to their vascularization capacities.

To demonstrate the relationship between angiogenesis and osteogenesis of CaP ceramics, in our previous work, four types of porous CaP ceramics, i.e. hydroxyapatite (HA), biphasic calcium phosphates (BCP-1 with HA/β-TCP≈70/30 and BCP-2 with HA/β-TCP≈30/70) and β-tricalcium phosphate (β-TCP), were investigated in terms of their vascularization and osteogenesis [9, 24]. The results revealed that only after 4 weeks of *in vivo* implantation into the thigh muscles of mice, the mature vascular networks had been formed within the ceramics. The vascularization could be due to the up-regulated expression of vascular endothelial growth factors (VEGF) and the enhanced proliferation of vascular endothelial cells (VECs) under the stimulation of CaP ceramics [24]. However, the previous works had not explicated the origin of the VECs. Besides the ingrowth of blood vessels from the nearby tissues, is it possible that spontaneous formation of new blood vessels within the ceramics? As we know, after a biomaterial being implanted into the body, an inflammatory response happens, accompanying with the migration of first neutrophils and then macrophages from nearby tissues and the circulation system into the implanting position [25–28]. These aggregated inflammatory cells secret a host of chemokines such as interleukin-1 (IL-1), IL-6, IL-11, IL-18, tumor necrosis factor-a (TNF-a) and transforming growth factor-β1 (TGF-β1) [29–31], promoting the further recruitment of mesenchymal stem cells (MSCs) and other cells [24, 32–35]. As a multipotent stem cells that can differentiate into different cell types, depending on the cellular microenvironment [36, 37], is it possible that the MSCs differentiate toward VECs under the stimulation of CaP ceramics, thereby offering the source of VECs for the formation of new blood vessels?

So far, some previous reports indicated that MSCs could phenotypically and functionally differentiated into VECs under certain conditions. Peng and Chi [38] co-cultured bone marrow-derived mesenchymal stem cells (BMSCs) with human umbilical vein endothelial cells (HUVECs), and found that the direct cell–cell contact and talk between the two types of cells initiated the differentiation of BMSCs toward VECs. The mechanism may be closely related to the promotion of VEGF secretion after the direct cell–cell contact and the cell fusion. Silva *et al.* [39] constructed a chronic ischemic model of dogs and injected BMSCs (100 × 10^6^ cells/10 ml saline) intraperitoneally. At 60 days postoperatively, the histological analysis and immunohistochemical staining confirmed that the BMSCs differentiated into an endothelial phenotype, thereby enhancing the vascular density and improving cardiac function. Oswald *et al.* [40] cultured BMSCs in the presence of 2% fetal calf serum and 50 ng/ml VEGF, and observed a strong increase of expression of endothelial-specific markers including kinase insert domain receptor (KDR), FLT-1 and von Willebrand factor (vWF).

Therefore, to explore the vascularization mechanism of porous CaP ceramics after *in vivo* implantation, in this study, the possibility of the oriented migration of BMSCs toward the ceramics and the differentiation of BMSCs into VECs under the stimulation of the ceramics were evaluated via *in vitro* and *in vivo* models. As we reported before, after implantation into the thigh muscle of mice, BCP-2 showed better osteogenesis and vascularization among the used four CaP ceramics [9, 24]. Hence, BCP-2 (HA/β-TCP≈30/70) were used as the material model in this study. The migration and differentiation of BMSCs were carried out by using a transwell model and directly culturing BMSCs on the ceramics under an *in vitro* or *in vivo* physiological environment, respectively.

## Materials and methods

### Materials

BCP-2 ceramic (HA/β-TCP≈30/70) was used for the *in vitro* and *in vivo* experiments, which was fabricated according to our previous method [9]. In brief, BCP precursor powders synthesized by wet precipitations were made into the slurries, which were foamed by H_2_O_2_ and then poured into the special mould. After rapid drying at 180°C in an oven, the green bodies were sintered at 1100°C for 2 h in a muffle furnace and then cut into the discs (Φ14 × 1.5 mm for transwell model, Φ14 × 2 mm for *in vitro* cell culture) or cylinders (Φ2 × 3 mm for *in vivo* implantation). The round coverslips (Φ14 × 0.17 mm for *in vitro* cell culture, Φ3 × 0.17 mm for *in vivo* implantation) were used as the control materials. Prior to use, all the specimens were sterilized by γ-ray irradiation at the dose of 25 kGy.

### Isolation of rat BMSCs and flow cytometric detection of cell surface antigens

Rat BMSCs were isolated from bone marrow of the femurs and tibias of newly born SD rats, and cultured in α-minimum essential medium (α-MEM) (Gibco, NY, USA) supplemented with 10% fetal bovine serum (FBS, Gibco), 100 U/ml penicillin, and 100 μg/ml streptomycin [34]. The phenotype of isolated cells was characterized by analyzing the cell surface antigens CD29, CD34, CD45 and CD90. In brief, digested P2 BMSCs were rinsed in PBS and resuspended in PBS to get a cell suspension at density of 5 × 10^5^ cells/ml. The monoclonal antibodies (Biolegend, USA), i.e. CD29 (Alexa Fluor 647 anti-mouse/rat), CD34 (FITC anti-human), CD45 (Alexa Fluor 488 anti-mouse), and CD90 (Alexa Fluor 700 anti-human), were added separately, followed by 45 min of incubation in the dark at 37°C. Labeled BMSCs were rinsed by PBS, centrifuged at 200×g for 5 min and resuspended in PBS, and then were analyzed using a flow cytometer (Cytomics FC500, Beckman, USA). Unstained cells were used as the control.

### Transwell migration assay

Cell migration assay was performed using a transwell model with 8 μm pore membrane filters (Corning Inc., USA). BMSCs were grown to subconfluence (70%) prior to harvest by trypsinization and labeling with CellTracker green (1 μM, invitrogen, USA) for 1 h at 37°C. The fluorescently labeled BMSCs (1 × 10^5^ cells/ml, 200 µl per well) were seeded in the upper chamber of the transwell. Four types of materials were placed in the bottom chamber, i.e. BCP ceramic discs (Φ14 × 1.5 mm) seeded with macrophages, coverslips (Φ14 × 0.17 mm) seeded with macrophages, BCP ceramic discs without seeded cells, and coverslips without seeded cells. The macrophages were labeled by CellTracker CM-DiI with red fluorescence (1 μM, MAIBIO, China) and the cell seeding density was 1 × 10^5^ cells/well. After incubation for 3, 6, 12, 24 and 48 h, the specimens in the bottom chamber were carefully taken out to check the BMSCs migration using a confocal laser scanning microscopy (CLSM, TCS SP5, Leica, Germany). Each experiment was performed in triplicate.

### BMSCs culture *in vitro*

BCP ceramic discs (Φ14 × 2 mm) and round coverslips (Φ14 × 0.17 mm) were placed in 24-well plates. The BMSCs (P3) were harvested by treatment with a trypsin/EDTA solution after reaching confluence. The harvested BMSCs were resuspended in a Dulbecco’s modified eagle medium (DMEM, Gibco, USA) to prepare a cell suspension at density of 2 × 10^4^ cells/ml for cell seeding. 1 ml of the cell suspension was added dropwise to the BCP ceramic discs or coverslips. The cells on the specimens were cultured in DMEM supplemented with 10% FBS (Beijing Minhai Biotechnology, China) and 1% penicillin/streptomycin at 37°C in an atmosphere of 5% CO_2_. The media were replaced at the 1st, 4th, 7th and 10th days. All the supernatants were collected and stored at −80°C for subsequent analysis.

### Cell morphology and viability

Live/dead staining was carried out to evaluate cell morphology and viability by using the fluorescein diacetate (FDA, Topbio Science, China) and propidium iodide (PI, Topbio Science, China) according to the specification. After being cultured for 1, 4, 7 and 10 days, the samples were washed twice with warm PBS and incubated in serum-free DMEM containing FDA and PI for 15 min. The stained samples were observed by CLSM. In the live/dead staining, the live cells would be dyed green after reacting with FDA, while the dead cells would be dyed red after reacting with PI. Each experiment was carried out in triplicate.

### Cell proliferation assay

The proliferation of BMSCs cultured on BCP ceramics or coverslips were detected by an MTT assay. The cells were incubated with 0.5 mg/ml MTT for 4 h at 37°C. The liquid in every well was removed and then dimethyl sulphoxide (DMSO) was added to each well for dissolution of the produced purple formazan salts. The optical density (O. D.) values were measured at the wavelength of 490 nm by a multifunctional full wavelength microplate reader (Varioskan Flash, Thermo Scientific, USA). Each experiment was carried out in triplicate.

### Polymerase chain reaction (PCR) assay for *in vitro* specimens

The gene expressions of VECs-related characteristic factors including KDR, vWF, vascular cell adhesion molecule-1 (VCAM-1) and Cadherin 5 (CDH5) in the BMSCs grown on BCP ceramic discs or coverslips were analyzed by a real-time PCR (RT-PCR). After 1, 4, 7 and 10 days of *in vitro* culture, the total RNA of samples was extracted using an RNeasy Mini Kit (Qiagen, Germany). For conversion of the RNA to complementary DNA (cDNA), an iScript cDNA Synthesis Kit (Bio-RAD, USA) was used. The RT-PCR reaction was carried out using a CFX96 RT-PCR detection system (Bio-Rad, USA) with SsoFast EvaGreen Supermix (Bio-Rad, USA). The sequences of primers for KDR, vWF, VCAM-1, CDH5 and GAPDH genes (genus: rat) were given in Table 1. GAPDH was selected as the housekeeping gene to normalize the gene expressions. The ΔΔ*Ct*-value method was adopted to calculate the relative value of gene expression. Each specimen was analyzed in triplicate.

**Table 1:** The primers and probes for real-time PCR

Target	Forward primer	Reverse primer
GAPDH	CTCAACTACATGGTCTACATGTTC	CCCATTCTCGGCCTTGACT
KDR	GATGTTGAAAGAGGGAGCAAC	ACATAGTCTTTCCCAGAGCGG
vWF	ACTTTGAGGTGGTGGAGTCG	CCTGTTCCTGGTATGTGTGC
VCAM-1	CCCAAACAGAGGCAGAGTGT	AGCAGGTCAGGTTCACAGGA
CDH5	CATCGCAGAGTCCCTCAGTT	TCAGCCAGCATCTTGAACCT

### Quantitative assay of protein expression

After cultured for 1, 4, 7 and 10 days, the total intracellular and extracellular proteins in the BMSCs cultured on BCP ceramic discs or coverslips were collected. The intracellular proteins of the cells were extracted by a CytoBuster protein extraction reagent (Novagen, USA) according to manufacturer’s instruction. The extracellular proteins were obtained by collecting the supernatants after cell culture. Quantitative analysis for the secreted KDR, vWF, VCAM-1, CDH5 proteins was carried out by using an enzyme-linked immunosorbent assay (ELISA, Cloud-Clone Corp., USA). The total amount of intracellular and extracellular proteins was also assayed by a BCA Protein Assay Kit (Pierce, USA) and then used to normalize the protein expressions of the KDR, vWF, VCAM-1 and CDH5. Each experiment was carried out in triplicate.

### *In vivo* implantation

**Figure 1:**
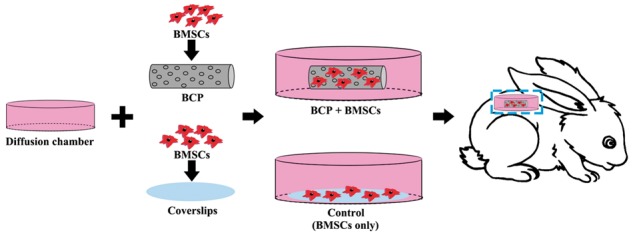
Schematic illustration of the *in vivo* model to analyze the differentiation of BMSCs under a physiological environment

To analysis the effects of BCP ceramics on the differentiation of BMSCs toward VECs in a real physiological environment, an *in vivo* model was established as shown in Fig. 1. The diffusion chamber was composed of a middle ring (Φ6.7 × 5 mm, cut from 96-well cell culture plate) and both ends of membranes (0.22 µm in pore size, Millipore, USA). The membranes were fixed on the ring by using DMSO as a binder. Prior to use, only one side of membrane was sealed to the chamber, which was sterilized by γ-irradiation. When reached 80% confluence, the BMSCs (P3) were trypsinized, counted, centrifuged and resuspended in DMEM to get a cell suspension at the density of 5 × 10^5^ cells/ml. The cell suspension (40 µl for each sample) was then added dropwise onto the BCP ceramic cylinders (Φ2 × 3 mm) or coverslips (Φ3 × 0.17 mm). The cell seeded BCP ceramic cylinders or coverslips were the carefully transferred to the chambers. The open side of each chamber was then sealed. The diffusion chambers were immersed in DMEM and then surgically inserted into a pocket subcutaneously at the back of adult New Zealand white rabbits under sodium barbital anesthesia. The wound was carefully rinsed with 0.9% saline solution and then closed with suture. All rabbits received ampicillins at consecutive 3 days postoperatively. The animal experiment was approved by the Animal Care and Use Committee of Sichuan University and operated based on the Guide for the Care and Use of Laboratory Animals published by National Academy of Sciences.

### SEM observation

At fourth day postoperatively, part of the diffusion chambers were retrieved. The specimens were fixed with 2.5% glutaraldehyde for 1 day, followed by dehydrated by an alcohol series (10, 20, 30, 40, 50, 65, 80, 95, 100%, 10 min each time) and xylene (100%, 30 min). After critical point drying, the BMSCs seeded BCP ceramics were cut in half along the long axis. The BMSCs on BCP ceramics or coverslips were observed by a field emission SEM (FE-SEM, S-4800, Hitachi, Japan).

### PCR assay for the *in vivo* implants

After 1, 4, 7 and 10 days of implantation, the diffusion chambers were harvested and the BMSCs seeded BCP ceramics or coverslips were retrieved. All the experiment procedures analyzing the expression of KDR, vWF, VCAM-1 and CDH5 genes were the same as those used for *in vitro* cell culture samples.

### Statistics analysis

All data were expressed as mean ± standard deviation, and every experiment was performed at least in triplicate. The statistical analysis was performed by a Student’s *t*-test, in which a one-way analysis of variance (ANOVA) with Tukey’s *post hoc* test was used. A statistical difference was considered as *P* < 0.05.

## Results

### Morphological observation and purity of BMSCs

**Figure 2:**
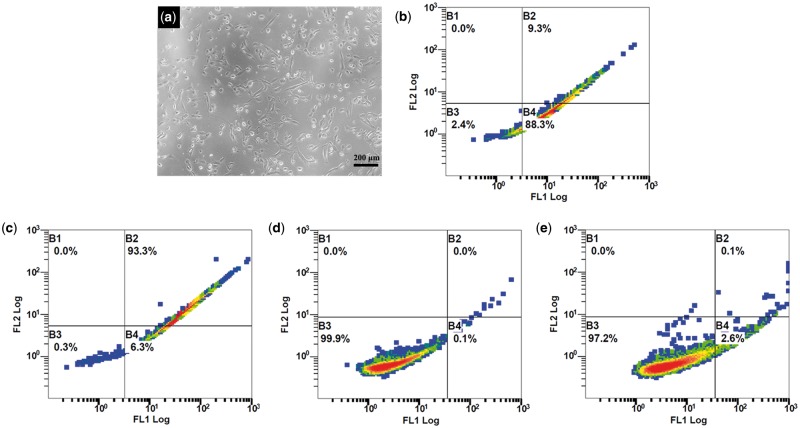
Representative images showing the rat BMSCs morphologies at the second passage (**a**), scale bar: 200 µm. Flow cytometric detection of cell surface antigens CD 29 (**b**), CD 90 (**c**), CD 34 (**d**) and CD 45 (**e**)

After 1 day of culture, primary cells attached, while most of the round cells with strong refractivity were erythrocyte. After attachment, BMSCs became rod-shaped or spindle-shaped. With the medium change, the majority of the non-adherent cells were eliminated and adherent cells (i.e. BMSCs) gradually proliferated. At passage 2, a proportion of BMSCs extended to become spindle-shaped, but others were mainly short rod-like, triangular or stelliform (Fig. 2a). The phenotype of isolated cells was characterized by flow cytometric detection of the lineage-specific markers, demonstrating that the cells expressed high levels of CD29 (97.6%, Fig. 2b) and CD90 (99.6%, Fig. 2c), while low levels of CD34 (0.1%, Fig. 2d) and CD45 (2.7%, Fig. 2e). The result confirmed that the isolated cells were mesenchymal types [41].

### Migration of BMSCs

**Figure 3:**
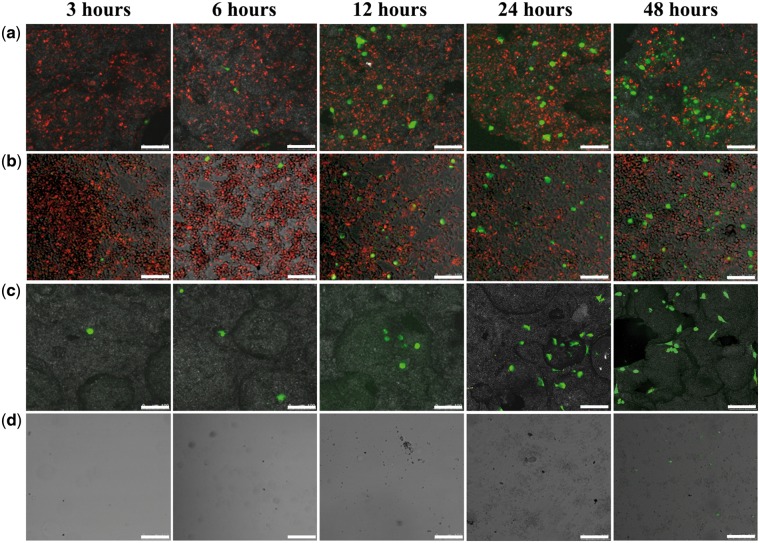
The migration of BMSCs in the upper chamber of the transwell inserts toward BCP ceramic discs seeded with macrophages (**a**), coverslips seeded with macrophages (**b**), BCP ceramic without seeding cells (**c**) and coverslips without seeding cells (**d**) in the bottom chamber of the transwell inserts

The migration behavior of BMSCs under different stimulation was carried out using a transwell model and observed by CLSM. The BMSCs were seeded in the upper chamber of the transwell inserts. In the bottom chamber, four types of materials were placed, i.e. BCP ceramic discs seeded with macrophages (Group A), coverslips seeded with macrophages (Group B), BCP ceramic discs without seeding cells (Group C), and coverslips without seeding cells (Group D). As shown in Fig. 3, after cultured for 6 h, the BMSCs in the upper chambers migrated to the bottom chambers of Groups A, B and C. After cultured for 12 h, the migration degree of BMSCs increased in an order of Group D < Group C ≈ Group B < Group A. After cultured for 48 h, the migration of the BMSCs in Group A was significantly higher than other groups, while the migration of BMSCs in Group C was slightly higher than that of Group B. Almost no migration of BMSCs was found in Group D. The results indicated that BCP ceramic could promote the directional migration of BMSCs, and the migration was more significant under the synergetic stimulations of macrophages and BCP ceramic.

### Morphology and viability of BMSCs cultured on BCP ceramic discs

**Figure 4:**
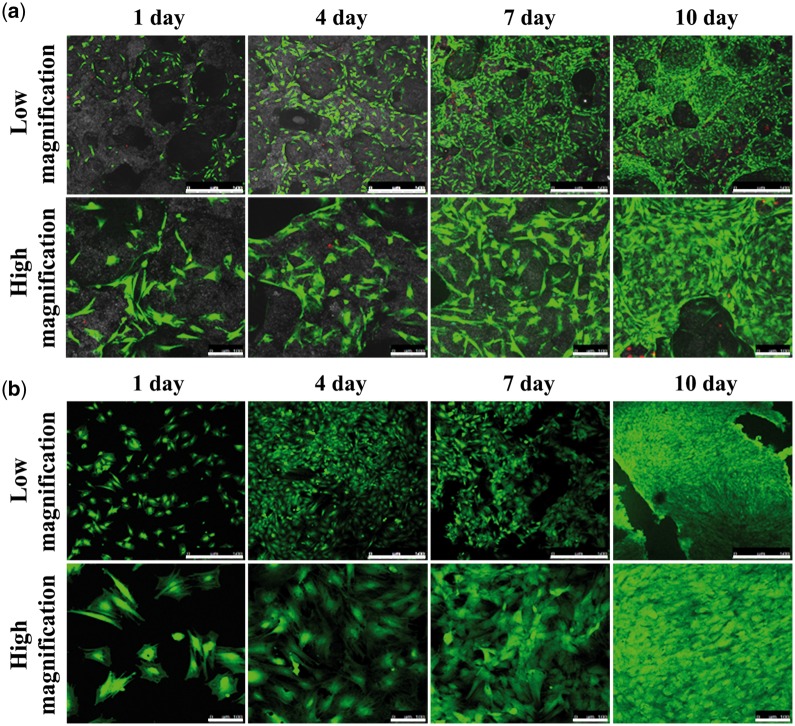
CLSM observation for BMSCs grown on BCP ceramic discs (**a**) and coverslips (**b**) at high and low magnification after 1, 4, 7 and 10 days *in vitro* culture. Scale bar for low magnification: 500 μm; for high magnification: 100 μm

To evaluate the effect of BCP ceramic on the cell morphology and viability, BMSCs were seeded onto the ceramic discs and cultured for 1, 4, 7 and 10 days. BMSCs cultured on coverslips were served as the control. The CLSM observation for the cell morphology and viability is shown in Fig. 4. With time prolongation, the number of the cells grown on BCP ceramic discs or coverslips both increased significantly, and few dead cells were found, indicating high viability of BMSCs and good biocompatibility of BCP ceramic. Besides, when compared with cells grown on coverslips, BMSCs grown on BCP ceramic showed more wide-spreading morphology, meaning that the ceramic supported the cell growth well.

### BMSCs proliferation

**Figure 5:**
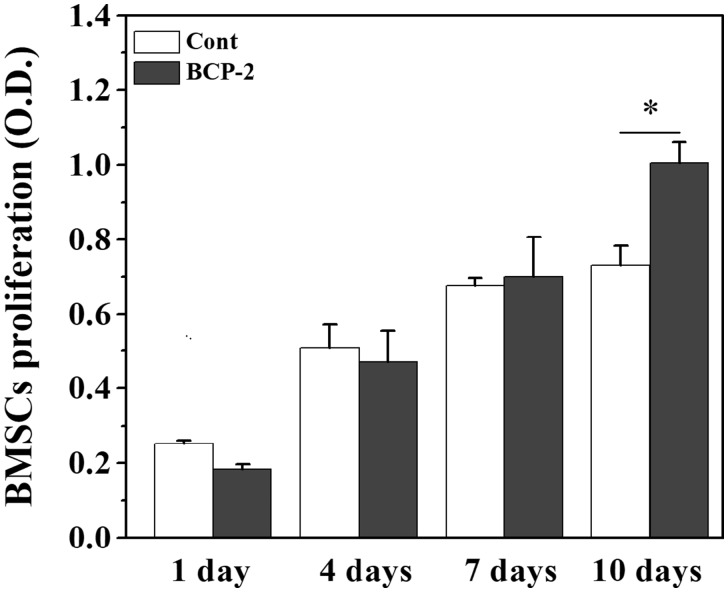
MTT assay for the proliferation of BMSCs on BCP ceramic discs and coverslips after 1, 4, 7 and 10 days *in vitro* culture. Data represent mean ± SD, *N* = 3. Significant difference: *P < 0.05

MTT assay was used to compare the proliferation of BMSCs on BCP ceramic discs and the coverslips. The results are shown in Fig. 5. The number of BMSCs grown on both BCP ceramics and coverslips increased gradually with time prolongation, which was well in accordance with the above CLSM observation. At the first 7 days, no significant difference in the cell proliferation rate was found between BCP ceramic and coverslip. After 10 days of culture, the cell number on BCP ceramic was significantly higher than that on the coverslip (*P* < 0.05). One probable reason was that the porous BCP ceramic provided a broader 3D space for cell growth, whereas the cells on the coverslip fell off or died since the space for cell growth was limited.

### Gene expression of VECs characteristic factors in BMSCs (*in vitro*)

**Figure 6:**
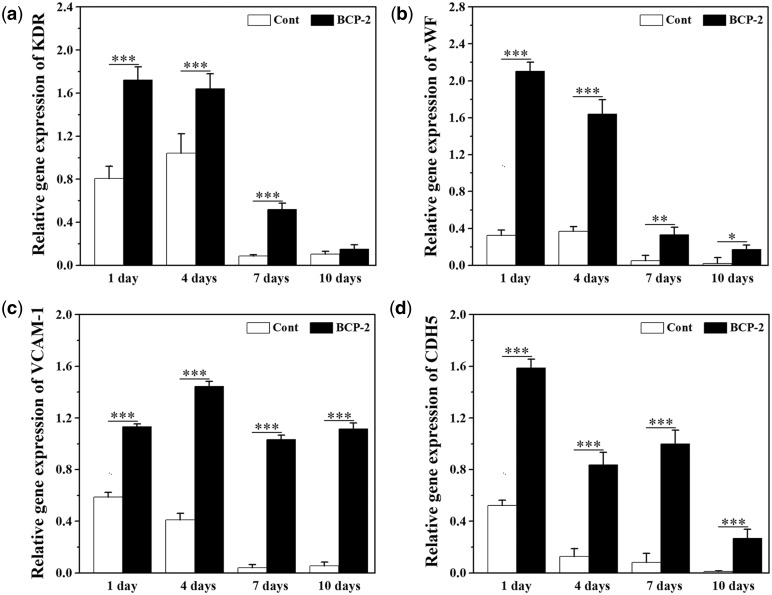
Expressions of KDR (**a**), vWF (**b**), VCAM-1 (**c**) and CDH5 (**d**) of BMSCs on the BCP ceramic discs and coverslips after 1, 4, 7 and 10 days *in vitro* culture. Data represent mean ± SD, *N* = 3. Significant difference: *P < 0.05; **P < 0.01; ***P < 0.001

Figure 6 shows the quantitative gene expressions of VECs characteristic factors (KDR, vWF, VCAM-1 and CDH5) in BMSCs after cultured on BCP ceramic discs for 1, 4, 7 and 10 days. The gene expression of cells cultured on coverslips was used as control. The gene expressions of KDR, vWF, VCAM-1 and CDH5 were all up-regulated by BCP ceramics when compared with control. For KDR and vWF, under the stimulation of BCP ceramics, the gene expressions showed the maximum at the 1st day, and then slightly decreased at the 4th day, following by significantly decreased at the 7th and 10th days. For VCAM-1, the highest gene expression was showed at the fourth day and then decreased slowly. For CDH5, the gene expression began to decrease after reaching the maximum at the first day, but there was a slight increase at the seventh day and then decreased again. The expressions of all the four characteristic factors were significantly higher than those of the control group, indicating that BMSCs differentiate toward endothelial cells under the stimulation of BCP ceramic at the gene level.

### Protein expression of VECs characteristic factors in BMSCs (*in vitro*)

**Figure 7:**
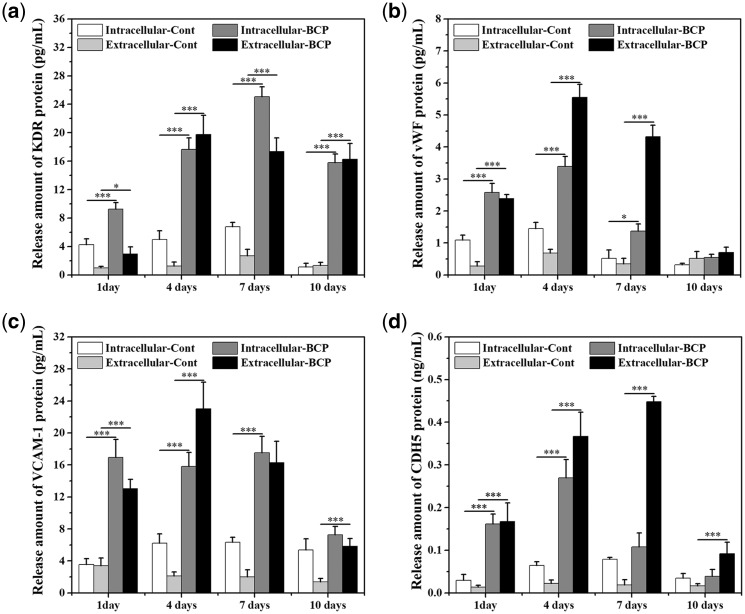
The intracellular and extracellular protein expression of KDR (**a**), vWF (**b**), VCAM-1 (**c**) and CDH5 (**d**) of BMSCs on the BCP ceramic discs and coverslips after 1, 4, 7 and 10 days *in vitro* culture. Data represent mean ± SD, *N* = 3. Significant difference: *P < 0.05; ***P < 0.001

The intracellular and extracellular protein expressions of VECs characteristic factors (KDR, vWF, VCAM-1 and CDH5) were analyzed by ELISA kit. As shown in Fig. 7, the protein expressions of KDR, vWF, VCAM-1, CDH5 were all up-regulated by BCP ceramic when compared with the control group. For KDR and VCAM-1 (Fig. 7a and c), under the stimulation of BCP ceramic, the intracellular expressions both reached the maximum value at the seventh day, whereas the maximum value of extracellular expressions appeared on the fourth day. For vWF (Fig. 7b), the intracellular and extracellular expressions both reached the maximum value at the fourth day, whereas the maximum value of extracellular expression appeared on the fourth day. For CDH5 (Fig. 7d), the intracellular expression reached the maximum value at the fourth day, whereas the maximum value of extracellular expression appeared on the seventh day. Similar to the results of gene expressions, the protein expressions of all the four characteristic factors were significantly higher than those of the control group, indicating that BMSCs differentiate toward endothelial cells under the stimulation of BCP ceramic at the protein level.

### Adhesion of BMSCs on BCP ceramic in diffusion chamber

**Figure 8:**
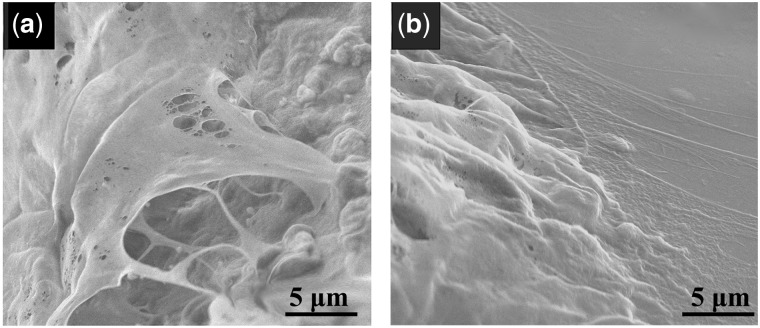
SEM images of the BMSCs seeded on the BCP ceramic discs (a) and coverslips (b) enclosed in the diffusion chambers after subcutaneously implanted *in vivo* for 4 days. Scale bar: 5 μm

The adhesion of BMSCs seeded on BCP ceramic cylinders or coverslips in the diffusion chamber after 4 days of *in vivo* implantation were checked by SEM (Fig. 8). It was observed that the BMSCs were well attached on BCP ceramic and coverslip under the real physiological environment, indicating that both materials had good biocompatibility and can support the adhesion and spreading of BMSCs.

### Gene expression of VECs characteristic factors in BMSCs (*in vivo*)

**Figure 9:**
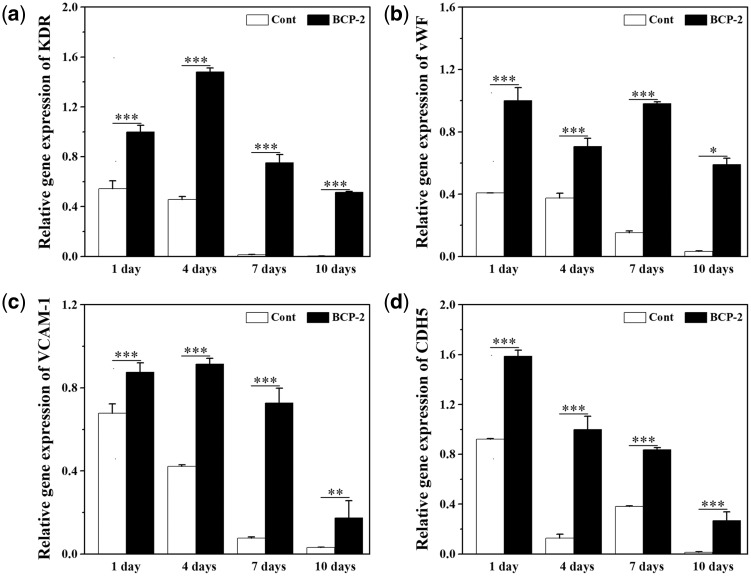
Expressions of KDR (**a**), vWF (**b**), VCAM-1 (**c**) and CDH5 (**d**) of BMSCs on the BCP ceramic discs and coverslips enclosed in the diffusion chambers after subcutaneously implanted *in vivo* for 1, 4, 7 and 10 days. Data represent mean ± SD, *N* = 3. Significant difference: *P < 0.05; **P < 0.01; ***P < 0.001

The *in vivo* diffusion chamber model was used to evaluate the possibility of BMSCs differentiation toward VECs under the stimulation of BCP ceramic in the real physiological environment. After 1, 4, 7 and 10 days of culture *in vivo*, the diffusion chambers were harvested and the BCP ceramic cylinders or coverslips were retrieved. The gene expressions of VECs characteristic factors in the BMSCs were analyzed by PCR assay, and the results are shown in Fig. 9. Similar to the *in vitro* results, the gene expressions of KDR, vWF, VCAM-1 and CDH5 *in vivo* were all up-regulated by BCP ceramic when compared with the control. For KDR and VCAM-1, under the stimulation of BCP ceramic, the gene expression showed the maximum value at the fourth day, and then slightly decreased. For vWF, the highest gene expression was showed at the first and seventh days. For CDH5, the gene expression began to decrease after reaching the maximum value at the first day. All the results demonstrated that BMSCs had phenotypically differentiated into VECs under the stimulation of BCP ceramic in the real physiological environment.

## Discussion

As a promising biomaterial used in bone tissue engineering, porous CaP ceramics present excellent osteoinductivity, but the mechanism is still not fully understood [9–20]. The relationship between vascularization and osteogenesis of CaP ceramics has been intensively investigated in order to reveal the mechanism [9, 20, 24, 42, 43]. In our previous work, the abundant vascular network formed in the inner pores of the implants after 4 weeks postoperatively when porous CaP ceramics were implanted in the thigh muscle of mice [9]. The rapid vascularization should be undoubtedly advantageous for new bone formation in the CaP ceramics, because it will bring about various functional cells, growth factors and other nutrient substances [44]. Thus far, it is still controversial that whether or not a biomaterial can induce angiogenesis. In fact, the formation of blood vessels requires a large amount of VECs. Moreover, it has been confirmed that CaP ceramics can promote proliferation and angiogenesis of HUVECs [24, 45]. At the early stage after a biomaterial implantation, the inflammatory response of the host leads to the aggregation of various inflammatory cells, MSCs and other progenitor cells at the implanting site. Therefore, we designed the *in vitro* and *in vivo* experiments by using two types of cells, i.e. macrophages and MSCs to verify if the CaP ceramics could recruit MSCs combing with macrophages and further stimulate their differentiation toward VECs, providing the cell source forming new blood vessels.

The migration of BMSCs was evaluated by using a transwell model. The transwell inserts with 8-µm pore membrane filter, which allows the nutrients, signaling molecules and proteins to pass through, while inhibits the transit of cells unless the cells become invasive in response to certain chemotactic cues. Under the stimulation of BCP ceramic, the BMSCs seeded on the upper chamber gradually migrated toward the material in the bottom chamber with time prolongation, and the macrophages seeded on the ceramic discs in advance further promoted the migration of BMSCs (Fig. 3a and c). As one of the most important inflammatory cells, macrophages can modulate a series of biological response by secreting a variety of inflammatory cytokines and growth factors, and these signalling molecules play a vital role in cellular processes involving in tissue regeneration [25, 29, 46–48]. The previous reports showed that macrophage cytokine secretion was highly dependent on the properties of the substrate materials [49–54]. Besides, CaP ceramics have strong protein adsorption ability, especially for some growth factors, such as bone morphogenetic protein 2 (BMP-2), VEGF, TGF-β1 and so on [9, 55–58]. Therefore, the synergistic stimulation of BCP ceramic and macrophages was stronger than that of the inert coverslip and macrophages (Fig. 3a and b).

To verify if the BMSCs can differentiate toward VECs under the stimulation of BCP ceramic, an *in vitro* co-culture of BCP ceramic and BMSCs was performed at first. The ceramic showed good biocompatibility and supported the cell growth well (Figs 4 and 5). After 1, 4, 7 and 10 days of culture, the PCR and ELISA analysis were made to evaluate the gene expressions and protein secretions of the characteristic factors of VECs, i.e. KDR, vWF, VCAM-1 and CDH5. When compared with the control group, all the four characteristic factors were significantly up-regulated by BCP ceramic at both gene and protein levels (Figs 6 and 7). Next, an *in vivo* diffusion chamber model simulating the real physiological environment, which allows penetration of the body fluids containing various ions, signaling molecules and other nutrients but prevents invasion of the foreign cells and the immune rejection by the host, was used for co-culture of BCP ceramic and BMSCs. After 1, 4, 7 and 10 days of *in vivo* culture, the BMSCs attached on BCP ceramic tightly and showed good spreading (Fig. 8). Similar to the *in vitro* results, the gene expressions of KDR, vWF, VCAM-1 and CDH5 were all up-regulated by BCP ceramic to different degrees when compared with the control group (Fig. 9). It is known that MSCs are multipotent stem cells and can differentiate toward various cell types, such as adipocytes, osteoblasts, chondrocytes, neuronal cells and so on, depending on the property of the substrate material and the cellular microenvironment [33, 34, 59–63]. Some previous reports showed that MSCs could differentiate into VECs under certain condition [38–40]. The results in this study give the sufficient proof that CaP ceramics have the potential to stimulate the differentiation of BMSCs into VECs.

Our findings in this study may provide better support to reveal the mechanism of vascularization and osteoinduction of porous CaP ceramics. After implantation, the CaP ceramics mediated inflammatory response would firstly recruit the aggregation of macrophages from nearby tissues and the circulation system in the inner pores of the implant. Then, the macrophages secrete various signalling molecules under the stimulation of the ceramic, which could further recruit MSCs and promote their proliferation and differentiate toward VECs or osteoblasts based on the different implantation period and the cellular microenvironment around the implant. The VECs in the CaP ceramics participate the formation and arrangement of new blood vessels, which further promote the subsequent new bone formation, in other words, lead to the happening of osteoinduction by the ceramics.

## Conclusion

This study puts forward a possibility for the origin of VECs during the rapid vascularization process after *in vivo* implantation of porous CaP ceramics. According to the *in vitro* and *in vivo* experimental results, the synergistic effect of BCP ceramic with the seeded macrophages in advance promoted the directional migration of BMSCs toward the material. Then, the BMSCs were stimulated to differentiate toward VECs by the ceramic, which could participate the formation and arrangement of new blood vessels and further promote the new bone formation. This study could give some useful guidance to explain the rapid vascularization of CaP ceramics and provide the useful support to reveal the mechanism of osteoinduction by the ceramics.
